# Long-term Outcome of Globus Pallidus Internus Stimulation for Pisa Syndrome

**DOI:** 10.7759/cureus.3838

**Published:** 2019-01-07

**Authors:** Brian L Anderson, Robert Ziechmann, Xuemei Huang, James McInerney

**Affiliations:** 1 Neurosurgery, Penn State Health Milton S. Hershey Medical Center, Hershey, USA; 2 Neurosurgery, Temple University Hospital, Philadelphia, USA; 3 Neurology, Penn State Health Milton S. Hershey Medical Center, Hershey, USA

**Keywords:** pisa syndrome, deep brain stimulation, dystonia, parkinson’s disease

## Abstract

Pisa syndrome, defined as dystonia leading to lateral flexion of the spine, is an increasingly recognized complicating factor in the treatment of Parkinson’s disease (PD). Symptoms may persist despite medical therapy, or medical therapy may not be tolerated due to adverse effects. Here, we demonstrate the long-term efficacy of deep brain stimulation (DBS) at the globus pallidus internus (GPi) for the treatment of Pisa syndrome. One patient with Pisa syndrome and Parkinson disease underwent bilateral GPi DBS with computed tomography (CT)-and microelectrode-based guidance. Follow-up with neurosurgery and neurology was done over a four-year period. The patient’s axial deformity decreased from approximately 45 to 25 degrees, and he reported significant relief from back pain. Bilateral GPi DBS is a safe and effective option for Pisa syndrome in patients with PD.

## Introduction

Dystonia in the setting of idiopathic Parkinson’s disease (PD) is an infrequent and unfortunate complicating factor of treatment. Although idiopathic focal dystonia is described in PD, most cases of dystonia are observed in the setting of treatment [1]. The association of chronic medication use with dystonia may reflect an imbalance between dopamine-, norepinephrine-, and serotonin-mediated mechanisms regulating axial muscle tone [2]. Treatment-induced dystonia poses a significant challenge in terms of management. Discontinuation or reduction in the dose of an implicated drug is rarely an accepted strategy as off-treatment dystonia is common and can result in significant pain, as well as the return of other associated symptoms of PD [3]. Other pharmacologic options for management include the introduction of anti-cholinergic drugs, which have a wide range of adverse effects, and treatment with botulinum toxin [4].

Deep brain stimulation (DBS) has until recently been reserved for the treatment of primary dystonia syndromes. Previous series had shown good clinical outcomes for patients with a variety of etiologies of primary dystonia but not secondary dystonia [5,6]. Recently, however, DBS has been successfully used to treat secondary dystonia from cerebral palsy and haloperidol-induced tardive dyskinesia [7-9]. For patients with Parkinson’s-related dystonia, DBS has primarily been described in patients with camptocormia (excessive flexion of the trunk), with outcomes improving as patient selection has improved; the duration of dystonia seems to predict response to treatment [10,11]. Treatment has primarily been directed at the subthalamic nucleus (STN).

Pisa syndrome is another form of dystonia related to the adverse effects of Parkinson’s medications, defined by lateral flexion of the spine. It is uncommon, with an incidence recently estimated at 8.8%, but lateral flexion of the spine can become severe enough to cause secondary scoliotic deformity and associated postural instability [4].

## Case presentation

**Figure 1:**
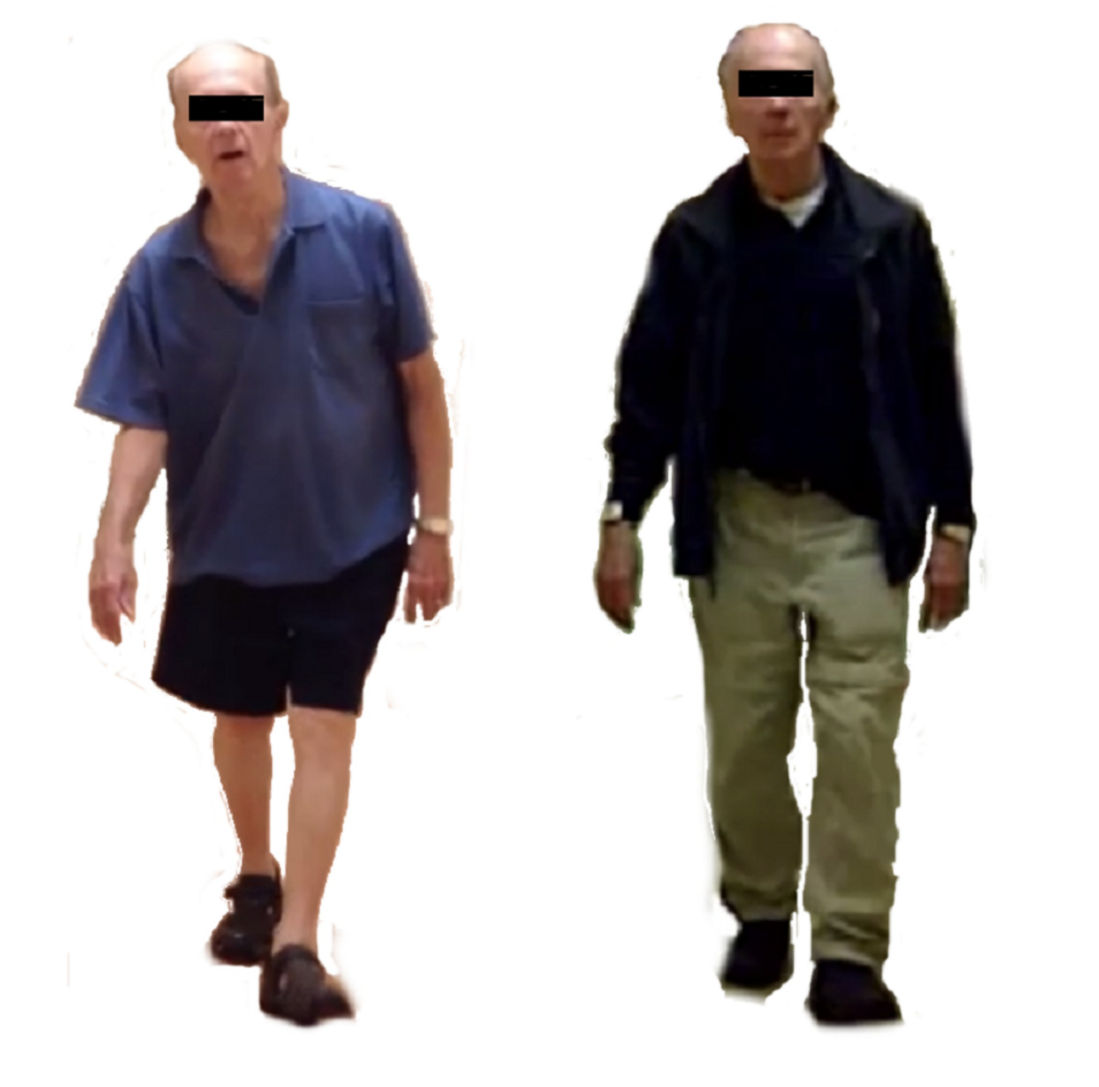
Before and after bilateral GPi stimulator placement. On left, three months prior to bilateral GPi stimulator placement. On right, three months following bilateral GPi stimulator placement. GPi: Globus pallidus internus

We present the case of a 73-year-old male with a history of mild scoliosis diagnosed with PD in 2007. Symptoms at the time of onset included decreased left-arm swing and mild bilateral upper extremity rigidity, with no evidence of postural deformity. Over subsequent years the patient developed mild bradykinesia, increased rigidity, hypophonia, and sialorrhea. The patient’s symptoms were well managed with medical therapy including pramipexole, rasagiline/selegiline, amantadine, carbidopa/levodopa. In 2012 the patient developed suspected Pisa syndrome with rapid onset of rightward leaning posture which did not improve with physical therapy, cessation of selegiline, or Botox injection. His postural changes progressed in severity which resulted in severe pain and decreased quality of life (Figure 1).

**Figure 2:**
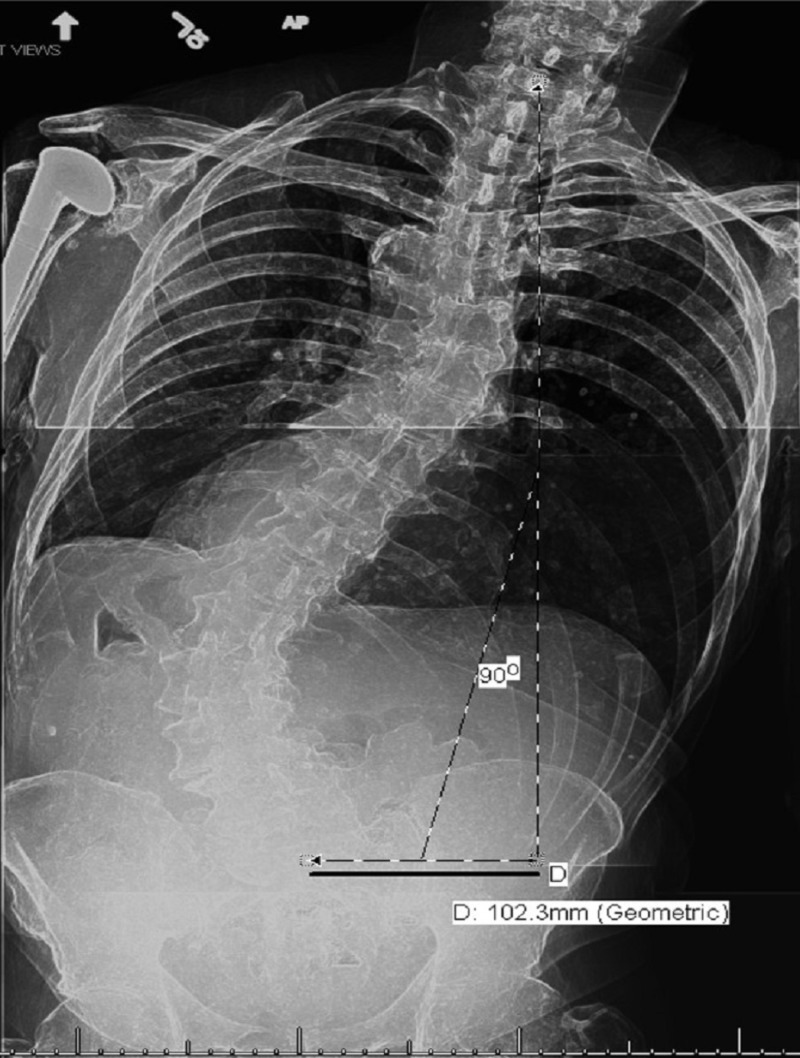
Erect scoliosis AP spine X-ray with coronal plumb line.

Spine X-rays showed interval increase in his thoracolumbar scoliosis (Figure 2).

The patient was evaluated for possible surgical correction of the deformity and was offered an extensive instrumented fusion. The patient was also evaluated for possible DBS as a means of treating his dystonia which was felt to be the source of his postural disorder. Given the evidence supporting globus pallidus internus (GPi) stimulation as a nondestructive treatment for dystonia and other Parkinson’s-related symptoms, he opted to proceed with DBS.

**Figure 3:**
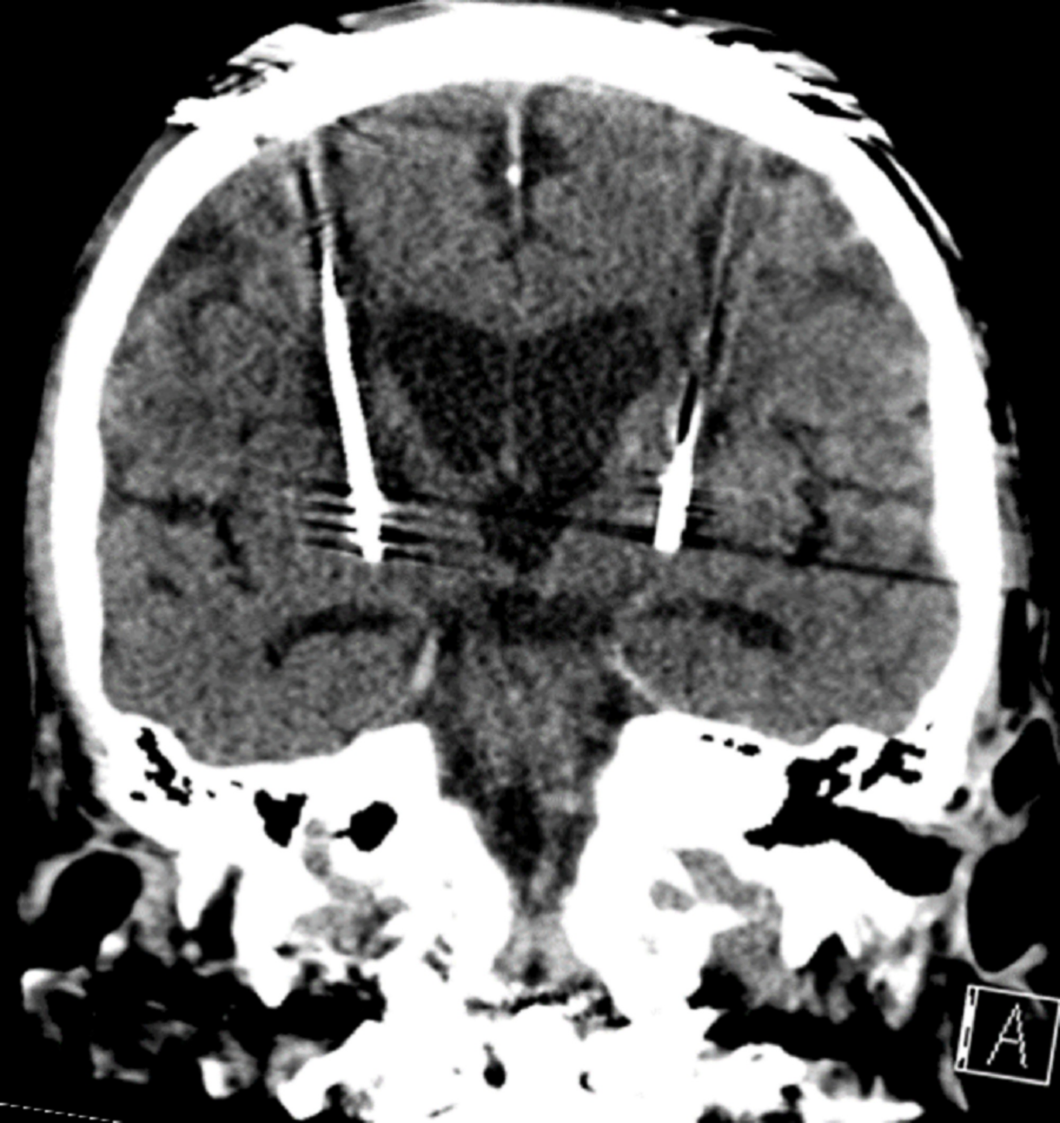
Computed tomography (CT) scan confirming placement of bilateral leads in the GPi (white arrows). GPi: Globus pallidus internus

The patient underwent bilateral GPi deep brain stimulator (DBS) placement and delayed pulse generator (IPG) placement. A Fred Haer Corporation’s WayPoint^TM^ Stereotactic System and STarFix^TM^ frameless stereotactic positioning platform were fixed to the patient using bone anchored fiducials. Using the microTargeting^TM^ Star^TM^ Drive system, microelectrode recording was performed. Target mapping was completed through the target with satisfactory findings. Intraoperative fluoroscopy was utilized to confirm placement, then the microelectrode was removed. The stimulating electrode was then placed and appropriate positioning was confirmed with fluoroscopy. Intraoperative testing was conducted for effectiveness and side effects. This was completed with good results. Confirmatory head computed tomography (CT) scan was obtained to ensure appropriate placement and evaluate for hemorrhage. This study confirmed appropriate placement and showed no unexpected changes. The patient returned to the operating room seven days later for the placement of the Medtronic Activa® Neurostimulator IPG. The cranial incision was again accessed and the leads recovered. The cranial leads were connected to intermediate wires which were tunneled to the IPG generator located just below the left clavicle. The leads were connected and diagnostic testing confirmed proper function. A confirmation head CT scan was obtained to ensure there was no intracranial lead migration (Figure 3).

His post-operative course was unremarkable and the patient returned for his initial IPG programming session approximately two weeks after placement. The patient did report a transient improvement of his symptoms following placement of the stimulators which was felt to be a lesion effect. At the time of initial programming, his symptoms were unchanged when compared to pre-operative findings. He again reported bradykinesia, rigidity, and significant rightward dystonic posturing with severe back pain and ambulatory difficulty. His axial deformity at this time was approximately 45 degrees. His medication regimen was unchanged throughout the perioperative period. He underwent a thorough programming session with maximum symptom relief achieved at a setting of C+/3-, pulse width of 90, rate of 130, and 3.5 volts on the left lead. The right lead was set to C+/11-, pulse width of 90, rate of 130, and 3.5 volts. The patient reported no significant side effects as a result of the programming.

The patient was seen at two-month intervals for assessment and to make minor adjustments to his IPG settings. The only change made to his initial settings was an increase in voltage from 3.5 to 5.0 volts bilaterally over multiple programming visits. Significant improvement was observed at his initial follow-up visit with a reduction in his rightward axial deviation. The patient continued to lean rightward but to approximately 25 degrees. He also experienced a significant reduction in his back pain. His ambulatory dysfunction was markedly improved with less difficulty rising from sitting, increased walking rate, and decreased postural instability. His bradykinesia and rigidity have also improved with placement of the GPi leads. Gradual improvement in his postural deformity has been observed over a four-year follow-up period. His postural deformity due to axial dystonia was felt to be resolved at seven months post-DBS placement. He maintained a slight rightward postural deviation which was essentially unchanged in comparison to his longstanding scoliotic deformity prior to the onset of PD and dystonia. No significant alterations were made to his medications during this follow-up period. In addition to the improvement of his dystonic symptoms, his steps-to-turn count was reduced from eleven to two, he no longer displayed a shuffling gate, and his finger and heel tap was essentially normal.

## Discussion

The successful use of GPi DBS in the treatment of secondary dystonia-induced postural deformity may offer a potential treatment strategy for this otherwise refractory condition. DBS of various targets, including the STN, pedunculopontine nucleus (PPN), and now GPi, have shown benefit in the treatment of Pisa syndrome in patients with Parkinson Disease [12-14]. Additional studies are needed to further analyze and delineate the effectiveness of stimulation at each target in this population. Although the effectiveness of DBS in treating secondary symptoms related to neuromodulatory medications has to date been limited, better outcomes may be seen with refined patient selection criteria [5,10,15].

## Conclusions

This report provides preliminary support to the use of pallidal stimulation in the treatment of uncommon postural disorders related to the management of PD. Successful treatment with GPi stimulation encourages continued research into determining the pathophysiology of these secondary symptoms with the purpose of providing better understanding and a more successful targeted therapy.
